# Supplementary material for: Matrix Metalloproteinase-3 is Key Effector of TNF-α-Induced Collagen Degradation in Skin
Source: Int J Mol Sci. 2019 Oct 22;20(20):5234. doi: 10.3390/ijms20205234 (PMC6829232; doi:10.3390/ijms20205234)
Supplement: Supplementary file 1 [file ijms-20-05234-s001.pdf]

Supplemental Figure S1

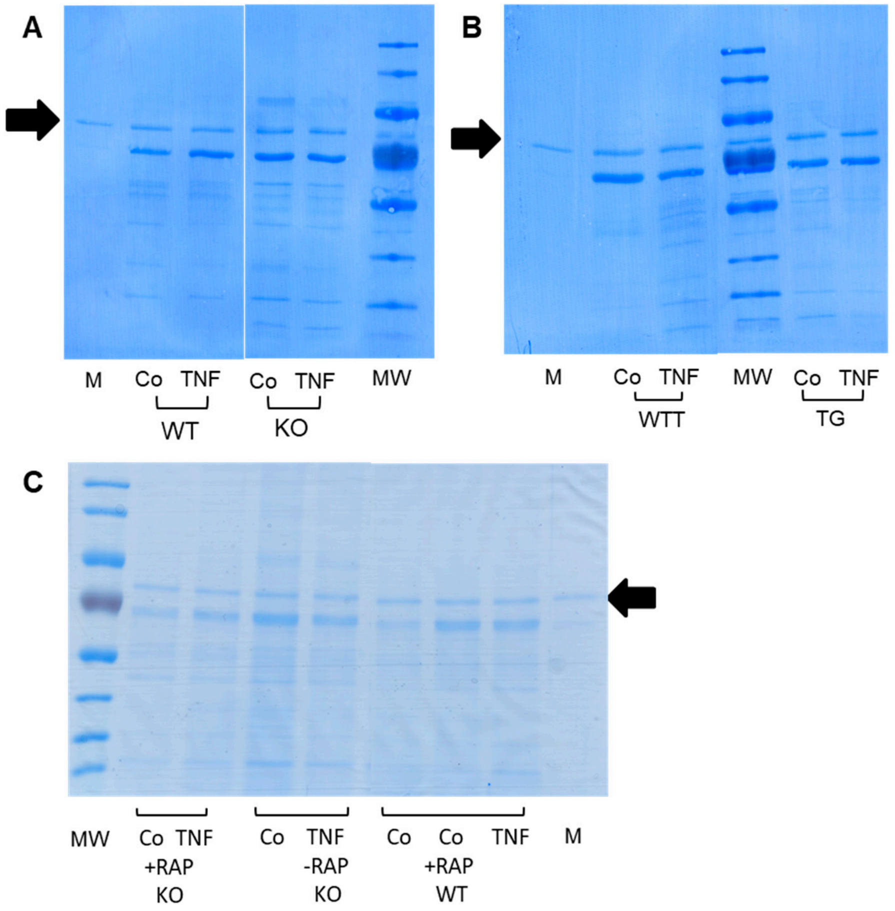

Supplemental Figure S1. Representative Coomassie blue-stained gels of conditioned media from KO and WT (**A**), and from WTT and TG skin explants incubated with or without TNF-α (**A**-**C**), and from KO and WT skin explants incubated in the absence (-RAP) or presence (+RAP) of RAP (**C**) for MMP-13 Western blot analysis. The Coomassie blue-stained gels were scanned and band densities determined for well loading according to the pooled control sample (M) consisting of control media. MW, molecular weight marker. Co, control treatment without additives; TNF, TNF-α (10 ng/ml). RAP, receptor-associated protein (250 nM). Arrow, pooled control band for calculation of sample loading. KO, MMP-3 knock-out; WT, wild-type control to KO; WTT, wild-type to transgenic mice (TG); TG, MMP-3 overexpression in skin.
